# Survival trends in primary myelodysplastic syndromes: a comparative analysis of 1000 patients by year of diagnosis and treatment

**DOI:** 10.1038/bcj.2016.23

**Published:** 2016-04-08

**Authors:** N Gangat, M M Patnaik, K Begna, A Al-Kali, M R Litzow, R P Ketterling, C A Hanson, A D Pardanani, A Tefferi

**Affiliations:** 1 Division of Hematology, Mayo Clinic, Rochester, MN, USA; 2 Department of Cytogenetics, Mayo Clinic, Rochester, MN, USA; 3 Department of Hematopathology, Mayo Clinic, Rochester, MN, USA

Myelodysplastic syndromes (MDS) are clonal hematopoietic stem cell disorders with significant clinical heterogeneity.^1^ Leukemic transformation (LT) rates and overall survival (OS) are extremely variable in MDS with the latter ranging from only a few months to almost a decade.^2^ As a result, treatment options vary from watchful waiting and supportive care to disease-modifying therapy including allogeneic bone marrow transplant.^2, 3^ Over the last decade, there have been three FDA-approved agents available for the treatment of MDS; 5-azacitidine was approved in 2004 for all subtypes of MDS;^4^ lenalidomide in 2005 for MDS with del(5q);^5^ and decitabine in 2006 for intermediate-/high-risk MDS.^6^ Lenalidomide is beneficial for only a subset of MDS patients with del(5q). In regards to the hypomethylating agents, 5-azacitidine and decitabine, a 9-month survival benefit has been demonstrated with 5-azacitidine alone based on results of the AZA001 clinical trial.^7^ On the other hand, none of the clinical trials conducted have demonstrated a survival benefit with decitabine therapy.^6, 8, 9^

In the AZA001 study, 358 high-risk MDS patients were randomized to receive 5-azacitidine with median survival of 24.5 months vs standard of care, which comprised a heterogenous group of patients treated with either best supportive care, acute myeloid leukemia induction chemotherapy or low-dose cytarabine with median survival of 15 months (*P*=0.0001).^7^ However, subsequent studies remain controversial regarding the ability of 5-azacitidine to improve survival outside of clinical trials.^10, 11^

The main objectives of our study are to (i) evaluate trends in OS and LT rate amongst primary MDS patients by year of diagnosis and additionally (ii) evaluate trends in OS by the treatments they received.

We utilized the Mayo Clinic database to identify patients with primary MDS during the time period January 1989 to May 2014 in whom bone marrow histologic and cytogenetic information was obtained at the time of diagnosis.^12^ World Health Organization criteria were used for MDS diagnosis and LT. A comparative analysis was performed based on the year of diagnosis commensurate with the approval of the aforementioned drugs (group 1: diagnosis prior to the year 2000, group 2: year 2001–2004, group 3: year 2005–2009 and group 4: year 2010–2014).

A total of 1000 patients met the above stipulated criteria. In total, 85% of patients were above 60 years of age (median 72 years) with 69% being males. The distribution of patients by the year of diagnosis was as follows: group 1 (*n*=281) 28%, group 2 (*n*=250) 25%, group 3 (*n*=264) 26% and group 4 (*n*=205) 21%. Median follow-up of our cohort was 27 months (range; 0–300 months) during which time 808 (81%) deaths and 129 (13%) LT were documented.

A comparison of patient characteristics by year of diagnosis was performed. Patients in groups 1 and 2 compared with groups 3 and 4 were more likely to present with anemia defined as hemoglobin <10 g/dl (61%/59% vs 50%/55%) (*P*=0.04). In addition, groups 1 and 2 displayed a higher incidence of refractory anemia (RA) (5%/4% vs 1% each), and RA with ringed sideroblasts (17%/16% vs 9%/8%), compared with groups 3 and 4 that had a higher incidence of refractory cytopenia with multilineage dysplasia (RCMD) (37%/44% vs 17%/28%) (*P*<0.001). The revised-International prognostic scoring system (IPSS-R) risk distribution was not significantly different; 17% very low, 36% low, 21% intermediate, 15% high and 11% very high risk with median survivals of 72, 43, 24, 18 and 7 months, respectively (*P*<0.001). As expected, a higher proportion of patients in groups 3 and 4 (41% and 57%, respectively) received 'disease-modifying' therapy, including allogeneic transplant and hypomethylating agents as opposed to only 6% and 22% in groups 1 and 2, respectively (*P*<0.001). Table 1 provides a summary of patient characteristics including treatment details and a comparison of patient groups by year of diagnosis.

Upon evaluation of the trends in OS and LT rate by year of diagnosis, we found that the median OS of the entire cohort was 30 months, with median OS and LT rates being similar among groups 1–4 at 31 vs 33 vs 30 vs 27 months (*P*=0.79) (Figure 1) and 10% vs 16% vs 12% vs 15% (*P*=0.25), respectively.

Subsequently, we analyzed the trends in OS by treatment received. In univariate analysis, we found survival to be significantly better in patients who underwent allogeneic transplant (*n*=65) with median survival of 55 vs 26 months for non-transplant patients (*P<*1.001); and among non-transplant lenalidomide-treated patients (*n*=44) with median survival of 54 vs 26 months for the remainder of patients (*P*=0.02). However, these results lost significance on multivariable analysis with the addition of age as a co-variate for transplant patients (*P*=0.28), and IPSS-R as a co-variate for lenalidomide-treated patient (*P*=0.10). Excluding transplant patients, patients who received hypomethylating agents (*n*=158) had similar survival to patients not treated with hypomethylating agents (27 vs 29 months; *P*=0.19, age-adjusted *P*=0.11). In addition, the 54 patients who received other chemotherapeutic agents that included cytosine arabinoside, idarubicin, daunorubicin, arsenic trioxide, all-trans retinoic acid or clinical trials had similar survival to patients not treated with these agents (33 vs 26 months; *P*=0.57, age-adjusted *P*=0.80). Supportive care alone was utilized in 702 patients that had comparable survival with the 298 patients who received 'disease-modifying' therapy (27 vs 34 months; *P*=0.05, age-adjusted *P*=0.11).

In conclusion, our single-center analysis of 1000 patients with primary MDS, stratified by year of diagnosis, shows that the poor outcome of these patients has not improved over the last two decades, inspite of the significantly higher utilization of 'disease-modifying' therapy, including hypomethylating agents since 2005. The lack of improvement in survival with hypomethylating therapy is consistent with recently published results from the Spanish MDS registry.^11^

However, our retrospective study is not designed to detect marginal survival benefit, which has thus far been reported in only one clinical trial.^7^

## Figures and Tables

**Figure 1:**
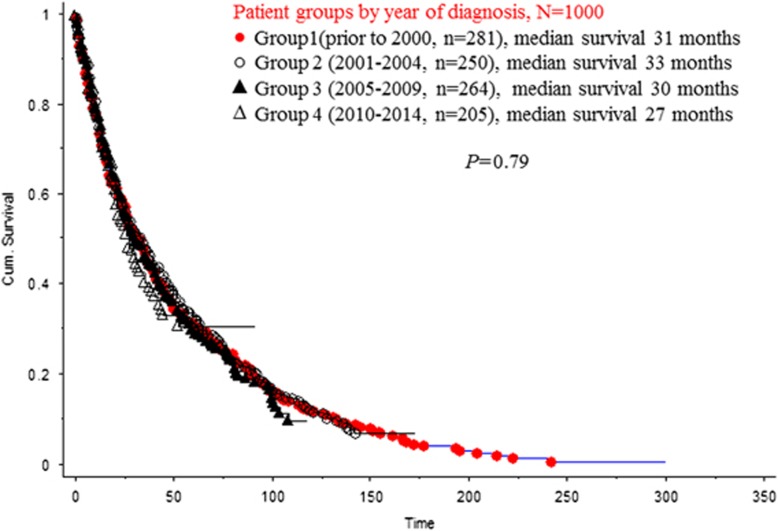
Survival trends among 1000 patients with primary MDS stratified by year of diagnosis.

**Table 1:** Clinical and laboratory characteristics with treatment details of 1000 patients with primary myelodysplastic syndromes (MDS) stratified by year of diagnosis

*Variables*	*Group 1 diagnosis prior to 2000 *(N=*281*)	*Group 2 diagnosis between 2001*–*2004 *(N=*250*)	*Group 3 diagnosis between 2005*–*2009 *(N=*264*)	*Group 4 diagnosis between 2010*–*2014 *(N=*205*)	P*-value*
Age in years, median (range)	72 (23–90)	71.5 (24–98)	72.5 (18–96)	74 (32–95)	0.18
Age >60 years, *N* (%)	239 (85)	210 (84)	226 (86)	175 (85)	0.96
Hemoglobin g/dl, median (range)	9.5 (5.8–13.6)	9.6 (6.2–14.6)	9.9 (6.9–15.7)	9.6 (5.4–15.7)	**0.04**
Hemoglobin <10 g/dl, *N* (%)	171 (61)	148 (59)	132 (50)	113 (55)	0.06
Absolute neutrophil count, median (range)	1.79 (0–22.6)	1.80 (0–15.4)	1.64 (0–50)	1.50 (0–12.2)	0.30
Absolute neutrophil count <0.8 × 10^9^/l, *N* (%)	65 (23)	62 (25)	73 (28)	57 (28)	0.56
Platelet count, 10^9^/l, median (range)	119 (8–1804)	107 (2–819)	105 (4–800)	89 (5–1408)	0.11
Platelet count <100 × 10^9^/l, *N* (%)	124 (44)	116 (96)	127 (48)	111(54)	0.17
Bone marrow blast %, median (range)	3 (0–19)	2 (0–19)	2 (0–18)	3 (0–18)	<**0.0001**
*WHO classification*, N *(%)*					
Refractory anemia (RA)	15 (5)	10 (4)	1 (0)	2 (1)	<**0.0001**
Refractory anemia with ringed sideroblasts (RARS)	49 (17)	45 (16)	25 (9)	17 (8)	
MDS with isolated del(5q)	21 (7)	14 (6)	19 (7)	4 (2)	
Refractory cytopenia with multilineage dysplasia (RCMD)	47 (17)	79 (28)	98 (37)	90 (44)	
Refractory cytopenia with multilineage dysplasia and ringed sideroblasts (RCMD-RS)	3 (1)	3 (1)	11 (4)	2 (1)	
Refractory anemia with excess blasts-1 (RAEB-1)	32 (11)	37 (15)	38 (14)	40 (20)	
Refractory anemia with excess blasts-2 (RAEB-2)	36 (13)	34 (14)	44 (17)	42 (20)	
MDS unclassified (MDS-U)	78 (28)	28 (11)	28 (11)	8 (4)	
					
*IPSS cytogenetic categories*, N *(%)*					
Good	197 (70)	179 (73)	159 (60)	137 (66)	0.10
Intermediate	48 (17)	38 (15)	55 (21)	32 (16)	
Poor	36 (13)	33 (13)	50 (18)	36 (18)	
					
*IPSS-R cytogenetic categories*, N *(%)*					
Very good	15 (5)	16 (6)	12 (5)	7 (3)	0.08
Good	187 (67)	165 (66)	152 (58)	131 (64)	
Intermediate	48 (17)	38 (15)	55 (21)	32 (16)	
Poor	8 (3)	15 (6)	10 (4)	8 (4)	
Very poor	23 (11)	16 (6)	35 (13)	27 (13)	
					
*IPSS-R risk distribution*					
Very low	38 (13)	55 (22)	48 (18)	27 (13)	0.05
Low	110 (40)	78 (31)	101 (38)	70 (34)	
Intermediate	66 (24)	54 (22)	41 (16)	46 (22)	
High	37 (13)	42 (17)	41 (16)	32 (17)	
Very high	30 (11)	21 (8)	33 (13)	30 (15)	
Transfusion dependence, *N* (%)	95 (34)	81 (32)	88 (33)	64 (31)	0.94
					
*Treatment*, N *(%)*					
Supportive care only (transfusions or erythropoiesis stimulating agents)	263 (94)	196 (78)	155 (59)	88 (43)	<**0.0001**
Disease-modifying agents including allogeneic transplant	18 (6)	54 (22)	109 (41)	117 (57)	
Follow-up in months, median (range)	29 (0–300)	33 (0–173)	30 (0–118)	18 (0–91)	NA
Deaths, *N* (%)	264 (94)	224 (90)	213 (81)	107 (52)	NA
Leukemic transformations, *N* (%)	29 (10)	39 (16)	31 (12)	30 (15)	0.25

Abbreviations: IPSS-R, revised international prognostic scoring system; MDS, myelodysplastic syndromes; NA, not applicable; WHO, World Health Organization.

IPSS cytogenetic categories: good (normal, −Y, del(5q), del(20q); poor: chromosome 7 anomalies, complex (3 or more abnormalities); and intermediate: all others IPSS-R cytogenetic categories: very good (−Y, del(11q)); good (normal, del(5q), del(20q) and del(12p) as sole abnormalities or double abnormalities including del(5q)); intermediate (del(7q), +8, +19, i(17q) or any other abnormality not listed in the other risk groups); poor (−7, inv(3)/t(3q)/del(3q), double abnormalities including −7/del(7q) or complex with 3 abnormalities); and very poor (complex with >3 abnormalities). Bold values are statistically significant *P*-values.
